# Fostering trust and understanding in social and healthcare services with migrant-origin parents: qualitative focus-group interviews of foreign-born mothers living in Finland

**DOI:** 10.1007/s00787-023-02288-4

**Published:** 2023-08-25

**Authors:** Saija-Liisa Kankaanpää, Venla Lehti, Pia Solin, Essi Salama

**Affiliations:** 1 Finnish Institute for Health and Welfare, Mannerheimintie 166, 00271 Helsinki, Finland; 2 Department of Psychiatry, HUS Helsinki University Hospital, P.O. Box 250, 00029 Helsinki, Finland; 3 Child Psychiatry, Turku University Hospital and University of Turku, Kiinamyllynkatu 4-8, 20521 Turku, Finland

**Keywords:** Migrant, Refugee, Family, Parenting, Wellbeing, Cultural sensitivity

## Abstract

Migrant-origin families may have a pronounced need for psychosocial support and healthcare services, but they face barriers in using services. To ensure the timely use of services, it is important that families understand how service systems work and trust care providers. Thirty-two migrant-origin mothers living in Finland participated in five focus-group interviews and shared their wishes for social and healthcare professionals on how trust and understanding can be increased. The data were analyzed with Qualitative Content Analysis. We identified six main themes related to the use of services and professionals’ behavior. These were the need for professionals to justify and explain questions, to meet each family as individuals, the importance of non-verbal communication, the need to talk about racism and discrimination, the importance of cultural sensitivity in services, and a discussion of positive aspects of life after migration and children`s strengths. To foster trust and mutual understanding in social and healthcare services, professionals should be aware of potential cultural differences in family life, while avoiding pre-conceived ideas. Misunderstandings can rise from language barriers and unclear or intimidating questions. It is important that professionals explain what they do and justify why they might inquire about a family’s personal matters. Working with interpreters is necessary when professionals and parents do not share a common fluent language. Professionals should also pay attention to their non-verbal communication and to being friendly. Finally, it is important to show interest in families’ experienced hardships such as racism as well as their strengths.

## Introduction

Children’s wellbeing is important for all parents and caregivers regardless of their background. Families who migrate may have a more pronounced need for healthcare services and psychosocial support. In particular, children with a refugee background can face multiple challenges that pose a burden on their wellbeing, such as exposure to potentially traumatic experiences, impaired parenting, and discrimination [1]. Nevertheless, research indicates that the use of preventive and primary healthcare is less frequent among migrant-origin children and children of migrant-origin parents,^1^ whereas the use of non-voluntary and emergency services is more pronounced in these groups [2]. Problems in accessing and using services in a timely manner can lead to the accumulation and worsening of problems. For example, in Finland, children born to migrant-origin parents receive more diagnoses of developmental disorders than children born to Finnish-origin parents [3, 4]. Migrant-origin parents’ children are also placed outside the family home by child protection services twice as often as children born to Finnish-origin parents [5].

Research has identified several barriers for migrant-origin adults’ and children’s use of healthcare services. Both healthcare providers in high-income countries and migrant-origin patients have reported that these barriers include lack of culturally sensitive services,^2^ language barriers, patients’ concerns about privacy and confidentiality, and mistrust of using services [6–9]. Enablers to service use, on the other hand, include increasing professionals’ cultural understanding and building trust between professionals and families [6, 7]. Information on how to build mutual understanding and ease migrant-origin families’ mistrust is therefore highly relevant.

In this study, we report on migrant-origin mothers' expectations for social and healthcare professionals and how mutual trust and understanding can be increased.

## Methods

### Design and setting

This research was a part of a project titled National support system for refugee mental health work and knowhow dissemination (PALOMA2; 2019–2021), coordinated by the Finnish Institute for Health and Welfare. This project was funded by the EU’s Asylum, Migration, and Integration Fund, AMIF.

Research data consist of five focus-group interviews (FGIs) with the total 32 migrant-origin mothers in Finland. Participants were recruited via migrant associations, third sector actors, and Finnish-language classes in the capital Helsinki region. Most of the participants in each focus-group knew each other beforehand. Inclusion criteria were having migrated to Finland and having at least one underaged child. Participants’ residence status was not relevant for the study, and it was not required that they have personal experiences of using certain types of healthcare services. All interviews were conducted in October 2020. Participants received a gift card as a thank you for their time.

Background information was collected on participant country of origin, native language, age, education, and number of children. The participants had 1–5 children. More detailed background information by focus-groups is presented in Table 1.

**Table 1:** Background information on participants by focus-group

Focus- group number	Number of participants	Country of origin	Focus-group language and interpretation	Age (in years)	Education (*n*)
1	5	Somalia	Somali, interpreted	26–48	No formal education: 3 Primary school: 2
2	5	Russia, Syria, Iran	Finnish, no interpretation	22–42	High school: 1 Higher education: 4
3	7	Russia, Belarus	Russian, interpreted	31–50	Higher education: 7
4	9	Somalia	Somali, interpreted	24–36	No formal education: 5 High school: 1 Vocational training: 2
5	6	Iraq, Syria, Palestine	Arabic, interpreted	19–44	Primary school: 1 Higher education: 5

Four of the five FGIs were conducted via interpreters and one in Finnish. The interpreters were native speakers of Somali, Arabic, and Russian. They were commissioned through a Translation Agency, except for the Russian language interpreter who was a representative of the Russian migrant association. In the groups with interpreters, the interviewer asked questions in Finnish which were then interpreted to target language and the responses back to Finnish. The participants in the focus-group without interpreter were Finnish learners recruited via language classes, where their Finnish-language skills had been evaluated to be sufficient for the interview.

Each FGI lasted between 75 and 120 min and was recorded. Finnish-spoken parts were transcribed verbatim. Analyses were conducted in Finnish. According to Wilkinson (2011), a word-for-word transcription is adequate in studies that aim to collect information about participants’ opinions and viewpoints [10].

FGIs were piloted to evaluate a new interview outline, the Culturally Sensitive Interview on Parenting (CSIP). The CSIP is a semi-structured interview outline that aims to help build rapport and mutual understanding between professionals and patients concerning parenting and children’s issues. It can be used in social and healthcare services, such as child protection services or child psychiatry, and when encountering families with a migration background. During FGIs, participants were asked their opinion on CSIP interview outline (e.g., were the questions easy to understand and how they felt about them). In each focus-group, the interviewer explained the interview setting and purpose, namely that the researchers were interested in participants’ thoughts on what are good or suitable questions for professionals to ask when working with migrant-origin families. The specific questions of the CSIP interview outline that were discussed in the focus-groups varied from one group to another. A predefined interview guideline was not used. However, in all focus-groups, the participants started to discuss the use of healthcare services more generally and shared their personal experiences. In this article, we report the findings related to the more general themes. The evaluation of the CSIP has been described elsewhere in Finnish [11]. The CSIP interview outline can be accessed online for free in Finnish, Swedish, and English at https://www.julkari.fi/handle/10024/141674.

The focus-group interviewer was a psychiatric nurse and a family psychotherapist with extensive experience in working with migrant-origin families. The interviewer did not know the participants beforehand or provide any services to them. Three authors are clinicians and academic research fellows with professional experience on mental health care of migrant-origin individuals of different ages. One author is an academic research fellow in social sciences. All members of the study team are white and Finnish-born.

### Analyses

In an exploratory research setting, we wanted to know what migrant-origin mothers’ thoughts and wishes for social and healthcare professionals are and how mutual trust and understanding can be increased when working with migrant families. We analyzed the data using Qualitative Content Analysis (QCA), which allows for the gathering of new knowledge and insights. QCA is a widely used technique, especially in health studies, such as nursing and psychiatry [12, 13]. As a method of analysis, it is flexible to use and very suitable for sensitive issues [14–16]. In the form of either concepts or categories, the description builds up a model, conceptual system or map, or categories [14, 17]. As a result, a condensed and broad description of the phenomenon is reached.

The inductive QCA process was divided into three phases: preparation, organizing, and reporting the data [16]. In the preparation phase, we decided that the smallest unit of analysis would be a sentence. Three researchers (the first, third, and fourth authors) read and analyzed the data independently. Each researcher identified meaningful sentences from the data in line with the exploratory research question. We then condensed these meaningful sentences, units, into descriptive categories and created sub- and main categories. For example, the sentence: “*It is better to add also, that these questions are for everybody, and that you want to know more about our culture and nothing special*.” was first condensed as an “Intimidating question”, then situated in the subcategory of “Worries and intimidating questions”, and further in the main category of “The need for professionals to justify and explain questions”.

After organizing the data into sub- and main categories, we discussed the findings. We noticed that there were units and subcategories that could relate to several categories [14–16, 18]. Differences or changes, for example in labeling the category, were discussed [15] and final categorization was reached by joint discussions. Subgroup analyses were not performed. Authentic citations are used to increase the trustworthiness and transparency of the research [19].

### Ethical approval

The independent ethics committee (IEC) at the Finnish Institute for Health and Welfare (THL) granted ethical approval for the study. This study was performed in line with the principles of the Declaration of Helsinki. Participants received written information about the study either in Finnish or in their native language. They also received a written privacy notice and provided oral consent.

## Results

### Content analysis themes

Using Qualitative Content Analysis, we found six main categories on how social and healthcare professionals can foster trust and mutual understanding when encountering families with a migration background. Some of the main categories were further divided into subcategories.

The main categories and subcategories were:

1. The need for professionals to justify and explain questions 
  - Expectations related to different professionals
  - Worries and intimidating questions
  - Misunderstandings
2. Meeting each family as individuals
  - Avoiding prejudice and stereotypes
  - Language barriers
3. The importance of non-verbal communication
4. The need to talk about racism and discrimination
5. The importance of cultural sensitivity in services
  - Cultural differences in parenting
  - Professionals’ cultural sensitivity
6. Considering strengths and positive aspects of life in services

1. The need for professionals to justify and explain questions
  Participants reported that care providers should justify and explain why they ask different types of questions. Professionals in different settings often ask parents a variety of questions (e.g., as a part of clinical assessment or when gathering background information). Still, the rationale behind why different types of information is gathered may be unknown to parents. We categorized this main category into three subcategories according to why explanations are necessary.
  - Expectations related to different professionals
    According to participants, a professional’s role or professional education may affect what types of behavior or questions are expected from them. Physicians were strictly seen as experts of physical health, and therefore, questions related, for example, to the parents’ or family’s background were not considered appropriate for physicians to ask. On the other hand, the psychologists’ or social workers’ role was seen as more flexible and it was better understood if they would inquire, for example, about the family’s past experiences
    “*Who asks? If there is no reason, then nobody will really answer. But if it is—for example the child has a problem and the psychologist asks, then that is of course important.*”
    Participant, Russian-language focus-group
  - Worries and intimidating questions
    Questions related to the child were more easily understood and considered acceptable, whereas questions concerning parents and their background as well as the purpose of this type of information were less understood. For example, questions regarding a family’s migration background or parental education were considered intimidating or useless by some participants. One participant from the Arabic-language focus-group stated that she would feel that she has done something wrong if the professional would ask more information about her background or growth environment.
    To make questions less intimidating, participants suggested that professionals could explain, for example, that all parents are asked the same types of questions and that these questions are asked to better understand the family’s socio-cultural context or current life situation.
    *“It is better to add also, that these questions are for everybody, and that you want to know more about our culture and nothing special.*”
    Participant, Russian-language focus-group
    Some participants stressed that it would be good if the professional stated that answering questions is voluntary as the situation can be intimidating for some parents, especially those who have negative experiences of formal hearing situations or interrogations. Emphasizing the voluntary nature of answering questions would, therefore, make building rapport and trust easier.
    “*It can soften the situation, if there’s fear or something like it. So, you can choose not to talk, but if you talk, maybe we can help you more or in a better way.*”
    Participant, Russian-language focus-group
    Some themes, such as mental health or the use of mental health services, were considered stigmatizing by some participants. However, how intimidating these themes were considered varied between participants and between the focus-groups. For example, participants in the Arabic-language focus-group were worried that child protection services could take their children away if they hear anything at all about parents’ background or their life (e.g., lack of education or divergent upbringing practices). Similar fears about child protection services were not explicitly stated in other groups. All groups seemed to agree that asking about these themes requires special consideration and explanations.
  - Misunderstandings
    Besides the care provider’s professional role and potentially intimidating questions, explanations were also considered important. Information asked for, and the reasons behind asking for it, should be made clear. Concrete examples may be needed to explain abstract and open-ended questions. For example, one participant said that if there is a concrete problem to which a solution is being sought, then the questions should be concrete, too. Some abstract ideas and words, such as *position, relationship*, and *relations*, were hard to understand in the Finnish-speaking focus-group, and they needed to be rephrased.
  Inquiring about the parent’s subjective views was, in general, considered a positive thing, but if not explained, these types of questions can be easily misunderstood. For example, participants considered it unprofessional to ask the patient’s views on why they came to the appointment instead of reading the referral or patient records and preparing for the appointment themselves.
  *“If I have booked the appointment and have told the reason for it, why we came, so the doctor should look it up and know. I get angry if I am asked this.”*
  Participant, Somali-language focus-group.
  However, when explained why this type of information is asked, participants considered it good that the professional is interested in their views on the situation.
2. Meeting each family as individuals
  Participants hoped that they would be encountered as individuals in social and healthcare services. This main category consists of two subcategories: avoiding prejudice and stereotypes and language barriers.
  - Avoiding prejudice and stereotypes
    Participants wished to be met as individuals, and not as representatives of their nationality, mother tongue, religion, or ethnicity. They expressed worries that care providers have pre-conceived ideas of certain cultures or nationalities, and that these ideas affect how families are treated. Participants also expressed concerns discussing parenthood or raising children on behalf of their ethnic communities. For example, participants did not want to talk about child-raising practices common in certain geographical regions or cultures. As one participant stated:
    “*I can’t take responsibility for the whole community and the community cannot be responsible for me. And each family has their own way, their inner culture.”*
    Participant, Somali-language focus-group
    Worries of being seen stereotypically were also related to the experiences participants had with racism and having been misunderstood on previous occasions. Therefore, this category also relates to the importance of explaining and justifying questions, since some questions can be seen as racist or stereotypical if their meaning is not explained. Participants expressed concerns that they are asked certain personal things only because of their ethnicity or religion. For example, Somali-speaking participants shared their experiences on a physician known in the community who had asked Somali-origin families unnecessarily how many children they have and why they want to have so many children.
3. Language barriers
  In situations where professionals and families do not share a common, fluent language, language barriers can add to the worry of not being met as individuals. The participants said that without a common language, they cannot adequately explain their unique situations or wishes, which may lead to misunderstandings, suspicions, and stereotypes. As one participant explained:
  *“If the child’s accident would happen there at home, and then the next day the child has an appointment or you as a mother think that you will take to child to get treatment, then it is thought straight away that maybe it has been another adult, where has the accident come from, and there are a lot of suspicions, how you behave at home and—Then you live without language skills, things happen at school, at the yard, at daycare.”*
  Participant, Somali-language focus-group
4. The importance of non-verbal communication
  Some themes regarding the family’s past or current family life were considered too personal or racist if they are not justified or explained. However, the participants stressed that more important than the question itself is how it is asked. As one of the participants stated:
  *“It is very important, the transparency. And then the tone of voice. And the way someone looks at you tells a lot, too, how the contact happens with them. These things are very important.”*
  Participant, Somali-language focus-group
  Some participants said that they do not mind talking about sensitive issues, such as parent’s potentially violent thoughts toward the child. They stressed that difficult issues should be addressed in a direct manner, but this needs to be done in a friendly and calm way.
5. The need to talk about racism and discrimination
  Many participants had experienced racism and violent, life-threatening situations in Finland, and some had had previous, negative encounters with healthcare professionals. One participant shared her experience with a nurse who acted differently with her because of her background:
  *“… the nurse behaved in a racist manner and did not take these tests that you usually take during pregnancy.*”
  Participant, Arabic-language focus-group
  Participants expressed a wish to talk about these kinds of experiences with professionals but said that they have almost never been asked about such experiences when using social and healthcare services. However, some said that issues related to racism can be hard to discuss because of feelings of shame.
6. The importance of cultural sensitivity in services
  In many of the focus-groups, participants talked about cultural differences in parenting (Finland vs. country of origin) and stressed the importance of professionals’ cultural sensitivity.
  - Differences in parenting
    Cultural differences in parenting and raising children were considered very important themes, but also themes that can be hard to discuss if professionals are not aware of or interested in these differences. Some participants had experiences of professionals forcing them to do something differently, for example, pressuring them to change their children’s diets without wanting to know why the participants might prefer a diet more common in their country of origin.
    In some cases, such as in the quotation below, the participants said that they are sometimes seen as incompetent or ignorant parents if they behave or think differently than what is expected in Finland:
    “*Yes, we don’t like it when we are considered ignorant. And then, anyways, like nobody is trained in parenting, but every mother has the desire to develop their own child and—hopes for the best for them.*”
    Participant, Somali-language focus-group
    Some participants considered that family life and the upbringing of children is so different in Finland compared to their country of origin that it is impossible to compare them. One participant stated:
    “*There (in the home country) the children are very, they are of much help for tasks at home, so they help and are beside the family. But here it is completely different.*”
    Participant, Somali-language focus-group
    As one way to make it easier to talk about potential cultural differences, participants considered it important that professionals show interest in understanding matters from the parents’ point of view. For example, inquiries about what parents consider to be good child behavior or good upbringing were considered welcome.
7. Professionals’ cultural sensitivity
  Cultural differences in parenting, or how people behave in general, were considered especially difficult issues in cases where the professional lacks cultural sensitivity and awareness. One participant said that there are many healthcare workers “who do not know anything about our culture”. Some cases demonstrate how the lack of cultural sensitivity can lead to misunderstanding in healthcare and social services:
  “*If a person is sick and comes (to the appointment), and someone asks how are you, so in our culture you say everything is fine. Even if you are sick. And the doctor can wonder that what, if you are there and you are fine, why have you come to the appointment.*”
  Participant, Somali-language focus-group
  The participants hoped that professionals would have some knowledge about different cultures and that they would at the same time be interested in each family’s everyday life.
8. Considering strengths and positive aspects of life in services
  The last identified main category is composed of families’ wishes to express their strengths and positive aspects of life after migration. The participants talked about how life in Finland can be much safer than before and how mothers can have new and different opportunities, for example, in working life compared to their opportunities before migration:
  “*I think that I found myself in Finland. Because it is true that I graduated from the university in Iraq, but I could not work. But now I am studying, I go working at the same time.*”
  Participant, Arabic language focus-group
  Inquiring about a child’s strengths was also considered very welcome. The participants said that it can make parents reflect on what the child is good at and what the child can do, which can strengthen the image the parents have of their child.

## Discussion

Parents and children of migrant-origin face barriers in accessing social and healthcare services [2, 8]. Fear and mistrust and lack of culturally sensitive services can affect families’ willingness to use services and their effectiveness. We explored, in a qualitative focus-group setting, migrant-origin mothers’ thoughts on factors that can help build rapport and trust in healthcare settings. We identified six main categories that can foster trust and mutual understanding. These included, for example, the need to justify and explain questions, meeting parents as individuals, and the importance of cultural sensitivity.

### Avoiding misunderstandings and importance of non-verbal communication

For migrant-origin families, the service system in the country of residence can be unknown and many may experience racism, discrimination, or prejudice. Efforts should be made to ensure that parents have understood everything as intended. Misunderstandings can be common when discussing abstract themes, or if the professional does not justify and explain questions.

According to the results, physicians in particular may need to pay more attention to explaining questions and clarifying why different types of information are being asked. To many, a physician`s role was strictly seen as being an expert of physical health.

In situations where justifications or explanations to questions are not given, misunderstandings are likely to happen and questions can be seen as intimidating, too personal, or racist. Parents might fear that their capability to take care of their children is questioned because of their history or background [20, 21]. Some parents might also fear that child protection services will take their child away if they disclose information on their lack of formal education or hard past experiences.

According to participants, a key factor in creating a safe and open environment is non-verbal communication and meeting each family individually, without pre-conceived ideas [22]. It is important to be aware that if parents are afraid of disclosing certain types of information or if they feel the professional is impolite, prejudiced, or culturally insensitive, they are unlikely to talk freely or commit to treatment. A professional’s friendly smile, use of eye contact or tone of voice can create a relaxed atmosphere that does not resemble formal hearing situations with the police or migration officers. Besides friendliness, clinicians´ open-mindedness and curiosity to understand matters from the parents’ point of view were seen as important factors. When working with refugee-origin families, it is particularly important to create an environment suitable for discussing personal topics, such as family history or parents’ experiences. Research confirms that holistic approaches and family involvement are crucial factors when working with refugee children and youth [7].

### Considering cultural differences and respecting individuality

According to our results, migrant-origin mothers wish for professionals to be aware of potential cultural differences in parenting while not assuming anything based solely on the family’s ethnic, cultural, or religious background. Our data indicated that there were both between- and within-group variation. This highlights that migrant-origin mothers, or for example Somali-, Arabic-, or Russian-speaking mothers, are not homogeneous populations. Besides linguistic or ethnic background, other individual-level factors, such as, education, length of residence in the new home country, or contact with services, are likely to affect migrant-origin mothers’ views. Research shows that migrant-origin families benefit from parental support programs especially if they are flexible and adapted to parents’ individual needs [23, 24].

Parents, in general, hope that providers tailor support to their personal needs and hope for the development of a trusting relationship with healthcare professionals [25]. Adapting care and building rapport with families should thus not be seen as specific to migrant-origin families only.

For social and healthcare professionals in transcultural settings, it can be difficult to simultaneously accommodate several aspects and balance between patients’ different expectations [26, 27]. As mentioned by the participants, professionals should meet each family as individuals and simultaneously have knowledge on different cultures. Care providers should explain and justify questions while avoiding making parents feel ignorant or incompetent. The patients’ differing and sometimes conflicting expectations can pose challenges and uncertainties to professionals and service systems on how to treat culturally diverse patients and adapt care for them.

### Exploring hardships and strengths together

Showing interest in parents’ experiences and ideas is important for creating a safe and open environment. To be able to reflect on one’s life, the patient must feel that the professional is trustworthy, on their side and prepared to explore relevant themes together with them. According to our results, experiences of racism are a theme that migrant-origin mothers would particularly like to talk about in healthcare settings. Racism and discrimination can have long-lasting and wide-ranging effects on children’s and families’ health and wellbeing [28, 29]. When asking about racism, professionals can show that they recognize its occurrence and relevance while also showing an interest in families’ wellbeing and lived experiences.

In building rapport or when planning treatment, it is also important to inquire about families’ strengths. Life after migration can be challenging but also better than before, and these aspects should be assessed. Young people with a refugee background have expressed a wish that professionals would recognize their coping strategies and strengths rather than only focusing on their problems or trauma history [7]. In clinical settings with refugee families, their resilience should also be identified and promoted [30].

### Strengths and limitations

This study provides important and concrete information on how to enhance cultural sensitivity and foster trust in social and healthcare services with migrant-origin families. However, our data are limited to mothers. Although the largest foreign-language populations in Finland are included in this study (Russian-, Arabic-, and Somali-speakers) [31], the study and sample size were not representative of the foreign-born population in Finland. Therefore, generalizing the findings should be done cautiously. Another limitation is the lack of sub-group analyses because of the small sample size. Therefore, we are unable to discern whether some of the findings are more specific to certain ethnic or linguistic groups than others. This has the potential negative effect of presenting all migrant mothers as a homogeneous group, whereas in reality, there is variation both within and between different migrant-origin populations.

Focus-group interviews (FGIs) were carried out by one interviewer, while data were analyzed independently and jointly discussed by three researchers, limiting interview variation and increasing the reliability of the results [32]. Many of the categories and subcategories found in the study are in some way related to one another, and the categorization done in this study should not be seen as definitive or fixed. There might be several indirect ways different themes are interrelated that we did not discover. For example, the experiences the participants had of racism and discrimination (main category 4) could be linked to how intimidating they experienced certain themes in clinical settings (subcategory 1 b) or to how important they considered non-verbal communication (main category 3).

Interpreters were indispensable during the FGIs. The lack of professional interpreter in the Russian language focus-group can be considered a limitation. During one focus-group conducted in Finnish, language posed a challenge for the participants, and they were unable to express their thoughts freely. The discussions in Finnish, however, provided necessary information on the importance using interpreters regarding issues, such as parenting or wellbeing. Additionally, these discussions demonstrated how easily abstract questions or words can be misunderstood and how important it is to use plain language if an interpreter is not available.

## Conclusion

Many social and healthcare professionals face increasing numbers of culturally diverse families. Showing respect and empathy is the key in building trust regardless of the parents’ background. There are, however, several aspects that should be considered especially if the parents are of migrant origin. Professionals should be aware of cultural differences, but still meet each family individually without stereotypes.

Language barriers can cause or worsen misunderstandings, affect what type of information is disclosed and how explanations are understood, and hinder the formation of mutual trust. Professionals may need to pay a special attention to their non-verbal communication and to being friendly. It is important that professionals explain what they do and justify why they might inquire about families’ past or personal matters. When professionals and parents do not share a common fluent language, it is important to work with interpreters. Finally, showing an interest in families’ strengths and the positive aspects of life following migration, subjective views as well as acknowledging possible hardships (e.g., racism) can help families feel they are met holistically and enhance their trust in services.

In future studies, larger sample sizes with mixed methods could be useful for gaining more information on families’ subjective ideas on service use. Information specifically on mental healthcare services and mental healthcare professionals’ experiences would be important, as mental health issues can be particularly sensitive for families with a migration background [8]. Also, the perspectives of migrant-origin fathers and children’s own thoughts and experiences regarding their health and healthcare services should be better understood [33].

## Data Availability

Full interview data are not available for ethical reasons.

## Footnotes

1 We use the term “parents” when referring to children’s primary caregivers.

2 Cultural sensitivity can be defined as creating an open and safe environment for patients as well as a professional’s interest in understanding how professionals’ and patients’ views can be affected by their cultural background [34].
